# Nutritional composition of *fufu* analog flour produced from Cassava root (*Manihot esculenta*) and Cocoyam (*Colocasia esculenta*) tuber

**DOI:** 10.1002/fsn3.250

**Published:** 2015-06-10

**Authors:** Oluwaseun P. Bamidele, Mofoluwaso B. Fasogbon, Dolapo A. Oladiran, Ebunoluwa O. Akande

**Affiliations:** ^1^ Department of Food Science and TechnologyObafemi Awolowo UniversityIle‐IfeOsun StateNigeria; ^2^ OK SweetsOshodi Industrial EstateLagosNigeria; ^3^ Department of Food Science and TechnologyFederal Polytechnic OffaKwara StateNigeria

**Keywords:** Cassava, cocoyam, minerals, pasting, proximate composition

## Abstract

Nutritional properties of fufu analog produced from co‐processing of cassava and cocoyam were studied. Cassava and cocoyam were fermented for 72 h, dried to obtain fufu flour. Proximate, functional, minerals, antinutritional factor, pasting properties, and sensory evaluation of various samples were determined. The results revealed that the moisture contents of the samples showed significant difference from control with values between 6.50 and 7.30%. The protein contents (1.68–4.98%), ash (1.84–4.01%), and crude fiber (1.42–4.56%) showed significant increase with increasing level of cocoyam, while the crude fat and carbohydrate reduced with increase in cocoyam. The minerals also increased with increase in cocoyam level with sample E having the highest value of Magnesium (32.15 mg/100 g). The antinutritional factors were very low and the pasting properties revealed the importance of cocoyam in the fufu analog produced. In conclusion, fufu produced from co‐processing of cassava and cocoyam has more nutritional qualities than the common fufu made from cassava alone.

## Introduction

Cassava (*Manihot esculenta Crantz*) production in Nigeria has greatly evolved from being a subsistence crop to an industrial cash crop (Tonukari 2004). The sweet (*Palmata*) and the bitter (*Utilisima*) varieties have been used in traditional production of products such as *fufu*,* Lafun*,* Gari*,* Abacha,* and *Tapioca* (Okoro 2007). *Fufu* is a fermented cassava product which is traditionally produced and consumed in some West African countries especially in Nigeria, Ghana, and Cameroon (Obadina et al. 2006). The sour taste, flavor, appearance, and texture are generally recognized as the main factors that determines the acceptability of the product (*fufu*). The consumer considers the product best when it has a smooth texture, a characteristic sour aroma, and a creamy‐white color. Traditionally, *fufu* is sold as a wet paste and this renders it highly perishable with a short shelf life (Tomlins et al. 2007). This problem has been addressed with the production of *fufu* flour that can be easily reconstituted into a paste with hot water and this product has become increasingly useful in and outside of Nigeria (Tomlins et al. 2007).

Cocoyam (*Colocasia esculenta)* constitutes one of the six most important root and tuber crops in the world and it belongs to the family *Aracea* (Ndabikunze et al. 2011; Bamidele et al. 2014) . In 1998, 8.3 million tons of cocoyam was produced by Burundi, Cote d'Ivoire, China, Japan, Ghana, Philippines, Madagascar, Thailand, and Nigeria (FAO, 1998). Cocoyam grows from the fleshy tuber which is used mainly for food and it supplies digestible starch with various types of nutrients (Ndabikunze et al. 2011). The cultivation of cocoyam is still very low when compared to other roots and tubers in Nigeria despite its superior nutritional value (Agbemologe 2013), this is due to poor research and lack of information on its utilization (Aderolu et al. 2009). Food security in various countries where cocoyam is cultivated can be enhanced by making it into culturally familiar food forms which is acceptable. This study therefore aimed at producing *fufu* using cassava root and cocoyam tuber in order to promote the better utilization of cocoyam.

## Materials and Methods

### Materials

Freshly harvested cassava and cocoyam roots were obtained from the Obafemi Awolowo University Teaching and Research farm, Ile‐Ife Nigeria. The cassava roots were about 10 months old at harvest. The outer skin was brown, cortex was creamy, and the pulp was white. The roots were transported to the laboratory and processed immediately.

### Methods

#### Processing of Wet fufu analog Paste

The wet *fufu* paste was produced as described by Sanni et al. (1998). The cassava and cocoyam roots were manually peeled, washed, and cut into pieces of different sizes using a knife. Cassava and cocoyam roots were mixed in different ratios (100:0, 80:20, 60:40, 50:50, and 40:60). Sample A serves as the control which was 100% cassava, 80% of cassava tuber to 20% of cocoyam serves as sample B, 60% of cassava tuber to 40% of cocoyam serves as sample C, 50% cassava to 50% cocoyam serves as sample D, and sample E comprises 40% cassava to 60% cocoyam. The differently mixed ratios were steeped in water in a plastic bowl for 72 h. After that, the soft roots were taken out, broken by hand and fibers were removed by sieving. The sieving was done manually by washing the mass through a plastic sieve. The filtrate was allowed to sediment for 24 h in a large plastic bowl. After sedimentation, the water was decanted while the sediment (*fufu*) was dewatered. This was achieved by putting the mash into a jute bag and pressed with a hydraulic press and left overnight to remove excess water.

#### Preparation of dried fufu powder

Wet *fufu* analog paste (50% moisture content) was dried in a cabinet drier (LEEC Ltd, Colwick, Nottingham, UK) at 65°C for 8 h (Sanni and Akingbala 2000). After drying, the dried *fufu* cake was milled into fine powder using an attrition mill. The powder was stored in a polythene bag at room temperature for further analysis.

#### Proximate composition

Proximate composition of the *fufu* samples for moisture, ash, fat, and protein contents were determined using the Association of Official Analytical Chemists (2005) methods. Total carbohydrate content was determined by subtracting the addition of moisture, crude fiber, ash, protein, and fat from 100%.

### Functional property determination

#### Water absorption capacity

This was determined using the method described by Numfor et al. (1996). Distilled water 15 mL was added to 1 g of *fufu* flour in a preweighed centrifuge tube. The centrifuge tube and content was agitated on a STUART scientific orbital shaker (Redhill, Surrey, UK) for 2 min and centrifuged at 4000 g for 20 min on a STUART scientific, SPECTRA (Merlin 503, Redhill, Surrey, UK) centrifuge. The clear supernatant was discarded and the centrifuge tube was weighed with the sediment. The amount of water bound by the flour was determined by difference and expressed as weight of water bound by dry flour (100 g).

#### Swelling capacity determination

Swelling capacity was determined by modification of the Lin and Zayas (1987) method. Each sample (2 g) was dispersed in 40 mL distilled water. The resultant slurry was heated at a temperature of 70°C for 30 min in a water bath, cooled to room temperature, and centrifuged at 598* *rpm for 30 min. The supernatant liquid was decanted and the centrifuge tube was dried for 25 min at 50°C inside a hot air oven. The residue was weighed (W_2_). The centrifuge tube containing the sample alone was weighed prior to adding distilled water (W_1_).

#### Bulk density determination

Bulk density was determined using the gravimetric method as described by Okaka and Potter (1979). Each sample (10 g) was weighed into a 25 mL graduated cylinder. The cylinder was gently tapped 10 times against the palm of the hand. The bulk density was expressed as the sample per volume occupied by the sample.

#### Mineral analysis

The mineral analysis was determined by the method described by AOAC (2005). The samples were ashed (Lenton muffle furnace AF11/6) at 550°C. The ash obtained was boiled with 10 mL of 20% hydrochloric acid in a beaker and then filtered into a 100 mL standard flask. The filtrate was made up to the mark with de‐ionized water. The minerals sodium (Na) and potassium (K) were determined from the solution using the standard flame emission photometer. NaCl and KCl were used as the standards (AOAC 2005). Phosphorus (P) was determined calorimetrically using the spectronic 20 (Gallenkamp, UK; Kirk and Sawyer 2005) with KH_2_PO_4_ as the standard. Calcium (Ca), magnesium (Mg), and iron (Fe) were determined using an atomic absorption spectrophotometer (AAS, Model SP9, Pye Unicam Ltd, Cambridge, UK). All values were expressed in mg/100 g.

#### Anti‐nutritional factor determination

The anti‐nutrients Saponin, Tannins, Trypsin inhibitors, Phytate, Glycoside, and Oxalic acid levels in the samples of *fufu* produced were determined using the rapid test method of Association of Official Analytical Chemists (2005).

#### Sensory evaluation of the fufu

The *fufu* samples produced were subjected to a sensory test using 10 panelists. The products were rated in terms of appearance, taste, texture, aroma, and overall acceptability on a 9‐point hedonic scale ranging from 9 (dislike extremely) to 1 (like extremely) and the results generated were analyzed using analysis of variance (ANOVA).

### Statistical analysis

The data were analyzed using IBM SPSS version 20.0 (IBM SPSS Statistics for Windows, IBM Corp., Armonk, NY). The mean and standard deviation (SD) of the triplicate analyses were calculated. ANOVA was performed to determine significant differences between the means, while the means were separated using the least significant difference (LSD).

## Results and Discussion

### Proximate composition

Proximate composition of *fufu* analog flour from co‐processing of cassava and cocoyam tuber is shown in Table 1. The result showed an increase in proximate composition of all the samples except sample A which is the control (100% cassava *fufu*). The moisture content of all the samples were generally low with the values ranging between 6.50% for the control to 7.30% for sample E. These values are within the recommended standard for flours as it reduces microbial proliferation and enhances shelf life (Kuye and Sanni 1999). The increase in protein, ash, crude fiber and decrease in crude fat and carbohydrate content of all the samples followed the trend reported by Bamidele et al. (2014) on *gari* analog made from the same source cassava and cocoyam. The presences of cocoyam in various samples were responsible for the higher value recorded in protein for all as reported by Ojo and Akande (2013). Sample A has the least value of Protein (1.68%), ash (1.84%), crude fiber (1.42%) and highest value of crude fat (1.32%) and carbohydrate (87.24%). The other samples (B, C, D, and E) had values ranging between 3.52 and 4.96% for protein, 2.10–4.01% for ash, 2.56– 4.56% for crude fiber, while the carbohydrate contents are lower than the control (77.97–83.49%) and the crude fat has similar value with no significant difference (*P *<* *0.05). This result is in line with the report of Lewu et al. (2010) who reported that cocoyam contain low crude fat.

**Table 1:** Proximate composition of *fufu* analog flour produced from Cassava (*Manihot esculenta Crantz*) and cocoyam (*Colocasia esculenta*) (%)

Samples	Moisture	Protein	Fat	Ash	Crude fiber	Carbohydrate
A	6.50^a^ ± 0.41	1.68^a^ ± 0.30	1.32^b^ ± 0.17	1.84^a^ ± 0.15	1.42^a^ ± 0.20	87.24^d^ ± 0.52
B	7.12^b^ ± 0.15	3.52^b^ ± 0.21	1.21^a^ ± 0.10	2.10^b^ ± 0.12	2.56^b^ ± 0.14	83.49^c^ ± 0.30
C	7.24^b^ ± 0.20	3.79^b^ ± 0.12	1.19^a^ ± 0.25	3.11^c^ ± 0.12	3.21^c^ ± 0.22	81.46^b^ ± 0.32
D	7.25^b^ ± 0.16	4.51^c^ ± 0.24	1.20^a^ ± 0.34	3.76^c^ ± 0.11	3.96^d^ ± 0.10	79.32^a^ ± 0.10
E	7.30^b^ ± 0.13	4.96^c^ ± 0.15	1.20^a^ ± 0.22	4.01^d^ ± 0.23	4.56^e^ ± 0.20	77.97^a^ ± 0.26

Means in a column with the same letter are not significantly different (*P *<* *0.05). Mean of three replicates. LSD (Least Significant Difference).

### Functional properties

The functional properties results of the *fufu* analog are shown in Table 2. The water absorption capacity of the *fufu* flour ranged between 198.11 and 176.46 g/mL for samples B–E compared to control (Sample A) 210.51 g/mL. This showed a decrease in water absorption capacity of all the samples in respect to the quantity of the cocoyam substituted into cassava. Water absorption capacity is influenced by degree of disintegration of native starches as reported by Falade and Okafor (2013). The decrease recorded may be due to less water retention or less absorption power of cocoyam starch granules caused by tightly packed starch granules which prevent the penetration of water (Soni et al. 1985). This result is in line with the report of Bamidele et al. (2014), who reported a decrease in water absorption capacity of *gari* made from mixture of cassava and cocoyam. The swelling capacity of the *fufu* analog samples was similar to that of water absorption capacity for various samples with sample A having the highest value (255.24 g/mL) followed by sample B, C, D, and E (235.15, 220.21, 200.10 and 186.46 g/mL respectively). Ezeocha et al. (2011) reported that the entrapped water molecule of food substance (starch granules) will be useful in making the food sample swell. The trend in this result may be as a result of cocoyam starch granules which absorbs less water and therefore reduces the swelling capacity of the samples containing cocoyam, whereas the reverse was the case of the control sample (100% cassava) which contains loose structure of starch granules. This result is also supported by the findings of Bamidele et al. (2014). The bulk density of all the samples were in two groups with samples A, B, and C having values of 2.15, 2.05, and 2.02 g/mL respectively while values for samples D and E are 1.98 and 1.98 g/mL respectively. Bulk density determination depends on the heaviness of the solid sample which is the determining factor for packaging requirements for materials handling and application in the food industry (Ezeocha et al. 2011). The particle size of the samples showed no significant difference.

**Table 2:** Functional properties of *fufu* analog flour produced from Cassava (*Manihot esculenta Crantz*) and cocoyam (*Colocasia esculenta*) (g/mL)

Samples	Water absorption capacity	Swelling capacity	Bulk density
A	210.51^e^ ± 0.49	255.24^e^ ± 0.86	2.15^b^ ± 0.10
B	198.11^d^ ± 0.57	235.15^d^ ± 0.50	2.05^b^ ± 0.22
C	190.30^c^ ± 0.36	220.21^c^ ± 0.27	2.02^b^ ± 0.11
D	180.15^b^ ± 0.38	200.10^b^ ± 0.32	1.98^a^ ± 0.15
E	176.46^a^ ± 0.38	186.46^a^ ± 0.44	1.98^a^ ± 0.20

Means in a column with the same letter are not significantly different (*P *<* *0.05). Mean of three replicates. LSD (Least Significant Difference).

### Mineral Analysis

Table 3, shows the mean values of the mineral components of *fufu* analog flour produced from cassava and cocoyam tuber. Sample E (40% cassava and 60% cocoyam) was abundant in all mineral elements tested while the control sample A (100% cassava) had the least of all mineral elements; Potassium (0.27 ± 0.10 mg/100 g), Sodium (0.26 ± 0.02 mg/100 g), Calcium (1.12 ± 0.10 mg/100 g), Magnesium (1.30 ± 0.05 mg/100 g), Phosphorus (1.22 ± 0.04 mg/100 g) and Iron (0.17 ± 0.02 mg/100 g). This is in line with the report of Lewu et al. (2010) that cocoyam is rich in minerals compared to some other tubers and this is well indicated from the ash content. It was observed that the mineral elements increased with increase in cocoyam percentage in the samples. This result is in agreement with the report of Bamidele et al. (2014) for gari analog produced from cassava and cocoyam. Magnesium was the highest mineral found in all the samples (B, C, D, and E) with values ranging between 18.51 and 32.15 mg/100 g, Sodium was next with values between 8.41 and 22.22 mg/100 g and Calcium had values ranging between 8.77 and 14.86 mg/100 g. Iron was the least mineral found in the samples with values between 1.46 and 3.54 mg/100 g. Phosphorus and Potassium were also not in abundance in the samples. The result obtained in this report indicates that substitution of cassava with cocoyam increased the mineral composition of the resultant blends.

**Table 3:** Mineral composition *fufu* analog flour produced from Cassava (*Manihot esculenta Crantz*) and cocoyam (*Colocasia esculenta*) (mg/100 g)

Samples	K	Na	Ca	Mg	*P*	Fe
A	0.27^a^ ± 0.10	0.26^a^ ± 0.02	1.12^a^ ± 0.10	1.30^a^ ± 0.05	1.22^a^ ± 0.04	0.17^a^ ± 0.02
B	3.86^b^ ± 0.12	8.41^b^ ± 0.17	8.77^b^ ± 0.22	18.51^b^ ± 0.30	1.98^ab^ ± 0.11	1.46^b^ ± 0.23
C	5.04^c^ ± 0.14	12.16^c^ ± 0.30	12.24^c^ ± 0.50	25.23^c^ ± 0.42	2.02^b^ ± 0.12	1.96^c^ ± 0.33
D	8.08^d^ ± 0.21	18.22^d^ ± 0.15	12.80^c^ ± 0.17	30.56^d^ ± 0.31	3.56^c^ ± 0.22	2.86^d^ ± 0.20
E	8.66^e^ ± 0.41	22.22^e^ ± 0.23	14.86^d^ ± 0.16	32.15^e^ ± 0.23	3.89^d^ ± 0.32	3.54^e^ ± 0.10

Means in a column with the same letter are not significantly different (*P *<* *0.05). Mean of three replicates. LSD (Least Significant Difference).

### Antinutritional factor

The control sample (A) contained the highest value for all antinutritional factors with Saponin (0.05 mg/100 g), Tannin (0.03 mg/100 g), Trypsin inhibitor (0.03 mg/100 g), Phytate (0.02 mg/100 g), Glycoside (0.04 mg/100 g), and Oxalic acid (0.04 mg/100 g). The remaining samples contained lesser values compared with the control. The reduction in the antinutritional factor may be attributed to processing condition which allowed fermentation of the tubers (cassava and cocoyam) for 72 h during which most of the antinutritional factors may be lost in water. It was also reported by Ojo and Akande (2013) that soaking has an influence in reducing the antinutritional factor of food. All these antinutritional factors were found below the acceptable level (1%) which shows that the samples were safe for consumption. This result is consistent with the findings of Bamidele et al. (2014) The antinutrional factors results of all the samples are found in Table 4.

**Table 4:** Antinutritional factor of *fufu* analog flour produced from cassava (*Manihot esculenta Crantz*) and cocoyam (*Colocasia esculenta*) (g/100 g)

Samples	Saponin	Tannin	Trypsin Inhibitor	Phytate	Glycoside	Oxalic acid
A	0.05^d^ ± 0.01	0.03^c^ ± 0.01	0.03^c^ ± 0.01	0.02^c^ ± 0.01	0.04^d^ ± 0.01	0.04^d^ ± 0.02
B	0.02^c^ ± 0.02	0.02^b^ ± 0.01	0.01^b^ ± 0.11	0.01^b^ ± 0.01	0.02^c^ ± 0.01	0.02^c^ ± 0.12
C	0.01^b^ ± 0.02	0.02^b^ ± 0.01	0.01^b^ ± 0.10	0.01^b^ ± 0.01	0.01^b^ ± 0.02	0.02^c^ ± 0.08
D	0.00^a^ ± 0.00	0.01^a^ ± 0.04	0.00^a^ ± 0.00	0.00^a^ ± 0.00	0.00^a^ ± 0.02	0.01^b^ ± 0.10
E	0.00^a^ ± 0.00	0.01^a^ ± 0.01	0.00^a^ ± 0.00	0.00^a^ ± 0.00	0.00^a^ ± 0.01	0.00^a^ ± 0.00

Means in a column with the same letter are not significantly different (*P *<* *0.05). Mean of three replicates. LSD (Least Significant Difference).

### Pasting properties

The ability and the power of starch to absorb water and swell depend on the temperature used in pasting the starch. In the presence of water and heat, starch granules swell and form paste by absorption of water. Table 5 shows the pasting properties of the *fufu* analog flour. The pasting temperature for all the samples varied significantly (*P *<* *0.05) between 72.53°C (sample A) to 89.12°C (sample E). Dreher and (1983) reported that pasting temperature depends on the size of granules with small granules more resistant to rupture and loss of molecular order. This may be the reason for increase in pasting temperature of those samples that contained higher percentage of cocoyam since cocoyam is known to contain small starch granules (Falade and Okafor 2013). Peak viscosity ranged from 312.04RVU (sample A) to 372.17 RVU (sample E). Peak viscosity which revealed the maximum swelling of the flour prior to disintegration has also been described by Liu et al. (2006) as the equilibrium point between swelling and breakdown of the granules. The presence of cocoyam in the flour sample contributed to the peak viscosity of the sample (B, C, D, and E).

**Table 5:** Pasting properties of “fufu” analog flour produced from cassava (*Manihot esculenta Crantz*) and cocoyam (*Colocasia esculenta*)

Samples	Peak viscosity (RVU)	Trough (RVU)	Breakdown (RVU)	Final viscosity (RVU)	Set back (RVU)	Peak time (Min)	Pasting temperature (^0^C)
A	312.04^a^ ± 0.22	222.96^a^ ± 0.22	89.08^a^ ± 0.21	312.42^a^ ± 0.12	89.46^a^ ± 0.22	6.70^a^ ± 0.50	72.53^a^ ± 0.25
B	322.41^b^ ± 0.14	226.26^b^ ± 0.30	98.17^b^ ± 0.11	323.41^b^ ± 0.17	90.12^b^ ± 0.30	7.06^b^ ± 0.42	75.56^b^ ± 0.20
C	332.53^c^ ± 0.32	230.78^c^ ± 0.50	101.22^c^ ± 0.43	330.51^c^ ± 0.16	91.22^b^ ± 0.27	7.38^c^ ± 0.17	82.00^c^ ± 0.30
D	352.43^d^ ± 0.34	231.26^d^ ± 0.51	104.15^d^ ± 0.30	336.22^d^ ± 0.20	94.51^c^ ± 0.41	7.56^d^ ± 0.32	86.21^d^ ± 0.50
E	372.17^e^ ± 0.50	236.35^e^ ± 0.32	106.24^e^ ± 0.10	340.14^e^ ± 0.28	94.86^c^ ± 0.40	7.69^d^ ± 0.30	89.12^e^ ± 0.30

Means in a column with the same letter are not significantly different (*P *<* *0.05). Mean of three replicates. LSD (Least Significant Difference).

Breakdown viscosity is a measure of the resistance to heat and shear stress of the samples. This varied significantly (*P *<* *0.05) between 89.08 RVU (sample A) and 106.24 RVU (sample E). This shows the paste is resistance to disintegration in response to heat and shear stress. The lower the breakdown viscosity, the greater the resistance of the flour and this is expected of flour with lower peak viscosities. Cocoyam addition had a huge effect on the breakdown viscosity of the samples. Owuamanam et al. (2010) defined setback as the difference between the breakdown viscosity and the viscosity at 50°C. The setback helps to determine the tendency of starch to retrograde. The setback value of all the samples differ significantly between 89.46 RVU (sample A) and 94.86 RVU (sample E). The higher the setback value, the higher the tendency of retrogradation of such sample during cooling with fast staling rate. Sample A had lower setback value and this indicates that retrogradation will be lower compared to other samples. This may be due to the presence of cocoyam in the other samples. The peak time for all the samples ranged from 6.70mins (sample A) to 7.69mins (sample E) which is an indication of response of starch present in the flour to heating. The peak time was similar in all samples except that of sample A. The trough viscosity which indicates the minimum viscosity value helps in measuring the ability of the paste to withstand breakdown during cooling and this ranged from 272.96 RVU to 236.35 RVU from sample A to E. The final viscosity of all the samples was similar to that of sample A. The final viscosity increased slightly with increase in cocoyam percentage. This is an indication that the food substance will form a viscous paste or gel after cooking and cooling, and will be resistant to shear stress during stirring.

### Sensory evaluation

The sensory evaluation result is shown in Table 6. All the samples except sample D and E compared favorably with sample A. The value ranged between 5.76 and 7.21 for appearance, taste (5.50–7.00), texture (5.41–7.02), aroma (5.21–7.17), and overall acceptability (5.02–7.10). Sample A has the highest score ranging from 7.82 appearance, taste (7.50), texture (7.92), aroma (7.65), and overall acceptability (7.77). Sample D contained 50% of cocoyam to 50% cassava and this may be the reason for its low rating while sample E contained the highest percentage of cocoyam (60%). The common *fufu* in Nigeria is made wholly from cassava; the presence of cocoyam in higher percentage may lead to a change in sensory properties of the *fufu* analog produce.

**Table 6:** Sensory evaluation of *fufu* analog flour produced from cassava (*Manihot esculenta Crantz*) and cocoyam (*Colocasia esculenta*)

Samples	Appearances	Taste	Texture	Aroma	Overall acceptability
A	7.82	7.50	7.92	7.63	7.77
B	7.21	7.00	7.02	7.17	7.10
C	7.01	6.92	6.98	7.04	7.10
D	6.56	6.50	6.22	6.54	7.07
E	5.76	5.50	5.41	5.21	5.02

Means in a column with the same letter are not significantly different (*P *<* *0.05). Mean of three replicates. LSD (Least Significant Difference).

## Conclusion

This study revealed the importance of cocoyam in *fufu* production which provided its great utilization in food industries and in the world at large. Nutritionally, sample E (40% cassava and 60% cocoyam) has the highest proximate composition but fell short in consumer acceptance while sample C (60% cassava and 40% cocoyam) has good proximate composition and similar acceptance to control samples by the consumers. Co‐processing of cassava and cocoyam at 60% cassava and 40% cocoyam can be used in production of *fufu*.

## Conflict of interest

None declared.
